# Psychophysiological restorative potential in cancer patients by virtual reality (VR)-based perception of natural environment

**DOI:** 10.3389/fpsyg.2022.1003497

**Published:** 2022-10-10

**Authors:** Rui Song, Qujing Chen, Ying Zhang, Qing'an Jia, Hongyun He, Tian Gao, Ling Qiu

**Affiliations:** ^1^College of Landscape Architecture and Art, Northwest A&F University, Xianyang, China; ^2^Shaanxi Provincial People's Hospital, Xi'an, Shaanxi, China; ^3^Institute of Medical Research, Northwestern Polytechnical University, Xi’an, Shaanxi, China; ^4^Town Planning and Designing Institute, Xi’an, Shaanxi, China

**Keywords:** esophageal or gastrointestinal carcinoma, EEG, virtual reality, human well-being, interdisciplinary planning

## Abstract

The positive significance of nature to human’ self-reported well-being has been widely confirmed, but less attention has been paid to the study of cancer patients, as well as the role of time on the restorative effects. Therefore, using virtual reality (VR) and the inclusion of patients with esophageal and gastrointestinal cancer as participants, this study conducted indoor experiments to explore patients’ psychophysiological recovery through the perception of five different environmental types with three to five interventions per week. There were 63 participants selected from the People’s Hospital in Shaanxi Province. Depending on their psychophysiological state, they would participate in three to five interventions in a week to compare the number of interventions needed to achieve maximum restoration. The five environmental types utilized varied in land cover, vegetation structure, and landscape characteristics, and were identified as blue space (BS), open green space (OGS), semi-open green space (SOS), closed green space (CGS), and gray space (GrS). Before and after viewing landscapes, the changes of psychophysiological indicators were measured to explore the influence of different environmental types on participants. The results showed that the participants preferred and received the highest perceived restorative potentials in BS and lastly, GrS. The green and blue spaces measurably increased positive emotions and perceived restoration while a decreasing negative emotions and the heart rate (HR) compared with the GrS. Participants had the highest level of relaxation while their eyes were closed in the EEG baseline stage. Moreover, participants received the most relaxation when they contacted with nature three times a week, which indicated that excessive natural participation may not be conducive to the sustained development of cancer patients’ psychophysiological health. Instead of field appreciation, VR could be utilized to increase the access of cancer patients to nature and then be used as an approach to landscape interaction.

## Introduction

The preferences for and positive benefits of natural spaces for the health and well-being of human beings have been widely demonstrated in comparison to urban built spaces (Hartig et al., 2014; de la Barrera et al., 2016; Stigsdotter et al., 2017a). Green and blue spaces, as common forms of natural space and valuable natural resources in cities (Wang et al., 2019; Valente et al., 2020), can bring significant restorative qualities within an environment for their varieties of vegetation and balance of refuge and open scenery (Stigsdotter et al., 2017b). These natural spaces can also function to protect people from illness (Van den Berg et al., 2010). However, considering their attributes vary in type, size, color, and vegetation composition (Huang et al., 2020), it is an important task for landscape architects to identify to which extent different natural spaces can contribute to well-being (Huang et al., 2020), so as to design effective spaces to benefit human health (Olmsted, 1865).

Vegetation structure is the physiognomic character of spatial configuration in green spaces, which can be used as an important quantitative indicator in green space design. The dominant frameworks in nature research have pointed to the importance of spaciousness in nature which has been related to mental restoration (Ulrich, 1993; Kaplan, 1995). Houwelingen-Snippe et al. (2020) also showed that spacious scenes can elicit significantly higher human emotion and social aspiration. However, there is a lack of uniform quantitative classification criteria for the study of spatial configuration characteristics of restorative environments, resulting in uncertainty about what kind of structural components will be more beneficial to human health (Hoyle et al., 2017). Vegetation structure is the most referred to representative configuration in a natural environment setting (Gao et al., 2019b). Therefore, the integration of vegetation structure into the green space classification will be conducive to an effective quantitative design of restorative landscape.

Previous studies on the health benefit of green spaces have considered various respondents with different social characteristics, including park visitors (Qiu et al., 2013), self-reported stressed individuals (Gao et al., 2019a; Huang et al., 2020), college students (Jiang et al., 2019; Gao et al., 2019b), etc. However, as a typical group whose physical and psychological health is generally threatened, few studies have taken the significance of nature intervention on patients into account (Trostrup et al., 2019), especially cancer patients with mobility difficulties due to the medical treatments typically performed in hospital settings. Traditional medical treatments, e.g., hospitalization, chemotherapy, and repeatedly invasive procedures, although significantly prolonging the lives of many cancer patients and in some cases effectively curing them, these results could be accompanied with certain harmful side effects to the body and mind for cancer patients (Chirico et al., 2016). Mental health problems of patients diagnosed with physical diseases have posed a great challenge to health care, and studies have also found that most cancer patients are accompanied by mental health problems (Collins et al., 2011). Therefore, the development of medicine must be combined with social psychology in order to achieve both physical and psychological treatment. As a widely recognized means of psychological recovery (Nilsson et al., 2011), the natural environment intervention seems to be a fairly promising treatment with less adverse reactions. Therefore, an “add-on” treatment for cancer patients based on the intervention of nature could significantly contribute to the modern health care system (Thompson Coon et al., 2011). However, although healing landscapes and horticultural therapy have been used in healthcare (Chang and Chien, 2017), there is still a lack of studies on how patients prefer different landscapes and which types of landscapes are more conducive to their physical and mental restoration, which can provide quantitative theoretical basis and practical methods for the construction of cancer patient-oriented restorative environments.

Until now, most traditional social-psychological studies of patients focus on the self-reported psychological indicators (Trostrup et al., 2019). Attention Restoration Theory (ART) and stress reduction theory (SRT) bring great possibilities to the assessment of self-reported health benefits (Ulrich, 1993; Kaplan, 1995). Raanaas et al. (2012) claimed that cardio-pulmonary patients who were more likely to view panoramic outdoor natural environments had higher levels of self-reported health. Rosenberg et al. (2014) found that brief outdoor adventures had significant positive effects on the psychosocial functioning of adult cancer patients. However, the studies of physiological indicators on cancer patients are relatively scarce. Trostrup et al. (2019) who systematically reviewed the significance of nature on the psychological health of patients, called for further attention to the importance of physical health and the relationship of the two. Studies have shown that people detect visual and auditory signals through the external environment, which then cause physiological response such as autonomic nervous system and measurable changes in electroencephalography (EEG; Li et al., 2021). Many physiological indicators, such as the heart rate (HR; Yu et al., 2020), blood pressure (Lee et al., 2009), and EEG results (Chang et al., 2008; Aspinall et al., 2015; Gao et al., 2019b) have been commonly used in nature and human health studies. Therefore, exploring environmental restoration through physiological measurements has been gradually proven to be reliable.

It is worth noting that previous studies have declared the important effects of contact with the natural landscape for human beings under different time periods of intervention (Lyu et al., 2019), but there is a lack of relevant research with cancer patients, especially for long-term studies with serial nature interventions. Kaplan and Kaplan (1989) mentioned that, with the increase of time, the ability of human beings to inhibit distraction weakens. Therefore, the difference in restoration levels under varying degrees of nature intervention intensity should be taken into consideration as well.

In addition, virtual reality (VR) can be an effective technology utilized in rehabilitation (Shin and Kim, 2015) due to its provision of relaxation by introducing various scenarios (White et al., 2018), and it thus plays a significant role in the physiological and psychological indicator measurements in the process of cancer treatment. It is non-invasive (Schneider et al., 2011) and provides the respondents with continuous natural experiences (White et al., 2018), which has great application prospects for patients with mobility difficulties. Oyama et al. (1999) suggested that, during the course of chemotherapy, the appreciation of the natural landscape could reduce the negative mood, pain, and anxiety of the patients. However, studies based on VR intervention have less focused on these health benefits of natural exposure, and the relevant outcomes are needed to be scientifically measured (White et al., 2018). In this study, VR was thus introduced to provide a case for the psychophysiological health studies of patients with esophageal and/or gastrointestinal cancer. Considering the lack of psychophysiological recovery studies under different intervention periods for cancer patients, the following three main research questions were developed:

1. What is the most preferred type of environment of cancer patients based on a comparative analysis of the intervention group and the control group?
2. What are the differences in psychological and physiological restoration of cancer patients in the two groups among different types of environment?
3. What is the degree of psychophysiological restoration within cancer patients under multiple interventions in a week?

## Materials and methods

### Stimulus material

VR technology was used to provide photo-realistic scenes of various landscapes, and panoramic photos were therefore collected as visual stimulus materials. Considering that the participants viewed the panoramic photos indoors instead of participating in on-site investigation, it was critical that the photos be able to represent the typical landscapes and a sense of reality, i.e., authenticity.

**Figure 1:**
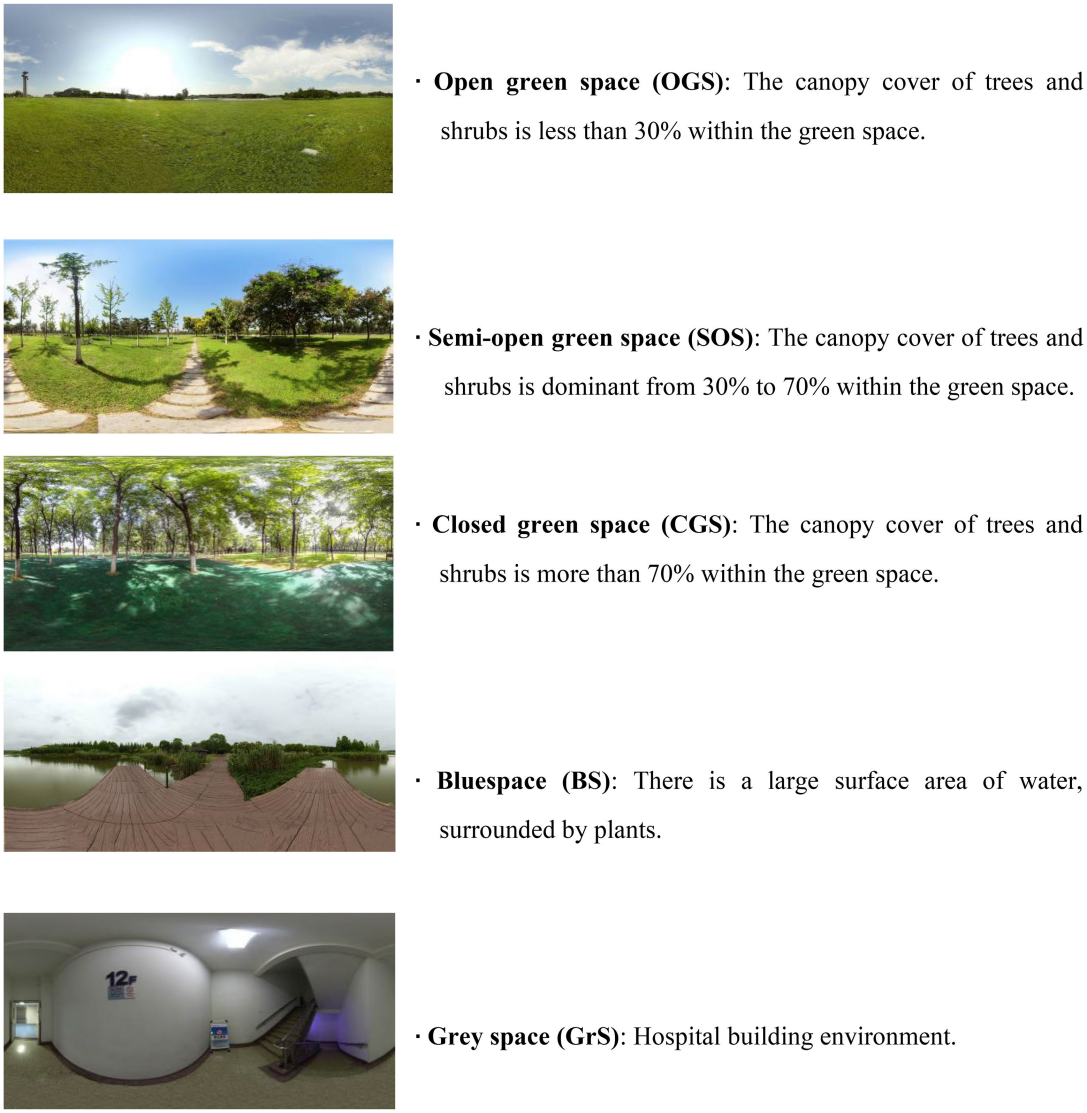
The representative panoramic photographs and description of the five typical types of environment identified by experts according to land cover, canopy cover ratios, size, location, species composition, setting configuration, and management regime. The intervention group viewed various green and blue spaces while the control group viewed the gray space.

To achieve the authenticity, the shooting venues selected as the randomized controlled stimulus materials were the city parks and the hospital respectively, places of which the cancer patients were familiar with and often visit. The panoramic photos were captured for the intervention group in the typical Chinese urban recreational forest parks which were the most welcomed by tourists with beautiful scenery on sunny days with no wind by an Insta 360 Pro-I panoramic camera with 7680*3840 (8 K) pixels in June 2018. Aerial planes were used to determine the canopy cover of the green spaces. The panoramic photos of the hospital indoor environment were also photographed for the control group. The shooting heights of the photos were adjusted to 1.6 m according to the average height of the human eyes in order to maintain authenticity (Jo and Jeon, 2020). There were 140 photos taken in total in the two groups. Nine expert landscape architects, ecologists, and silviculturists were invited to classify and select park photos through a visual and bio-physical characterization of the landscape. First, the photos were divided according to the land cover type into green space (GS) and blue space (BS). Then, the GS was subdivided into open green space (OGS), semi-open green space (SOS), and closed green space (CGS) based on the actual measurements of the canopy cover ratios of trees and shrubs (Figure 1). Next, the most representative photos of BS, OGS, SOS, and CGS were determined by the size, location, species composition, setting configuration, and management regime. Two tumor-related attending doctors and three head nurses were also invited to select the most representative photos of the indoor hospital environment as gray space (GrS). This selection process resulted in five typical types of environment (Figure 1). For each type of environment, five photos were included, and a total of 25 photos were selected, which were displayed in the VR equipment (Pico Goblin VR all-in-one, 2,560*1440 pixels resolution).

### Participants

After considering the increasing morbidity in the world and the high mortality rate in China (Anderson et al., 2019; Zhao and Lim, 2020), esophageal and gastrointestinal cancer patients were finally selected as the participants, as they fit the criteria and were willing to participate. The patients with esophageal or gastrointestinal cancer who met the inclusion–exclusion criteria (Table 1) were volunteered for the experiment by the People’s Hospital in Shaanxi Province, China. The inclusion–exclusion criteria were created by the tumor-related attending doctors, head nurses, and landscape architects. In total, 70 patients participated in the experiment based on the inclusion–exclusion criteria. Participants were randomly (single-blind) assigned into two groups, the intervention group and the control group. The intervention group was exposed to virtual green and blue spaces, and the control group was exposed to virtual gray space. As a result, 34 participants were in the intervention group and 36 in the control group. Seven respondents (2 in the intervention group and 5 in the control group) were excluded due to incomplete experimental data caused by discomfort halfway through the experiment. Therefore, 63 patients participated, including 32 participants in the intervention group (mean age = 61.31 ± 15.10, 23 males, 9 females) and 31 participants in the control group (mean age = 59.48 ± 12.06, 20 males, 11 females). For participants in the intervention group, their monthly income averaged less than 5,000 RMB (approximately equal to $712.5). Most of them had basic education (less than a senior high school education) and lived in a rural environment. The participants of the control group had similar social demographic information as those of the intervention group. All subjects gave their informed consent for inclusion before they participated in the study. The study was conducted in accordance with the Declaration of Helsinki, and the protocol was approved by the Ethics Committee of College of Landscape Architecture and Arts, Northwest A & F University.

**Table 1:** The inclusion–exclusion criteria that patients with esophageal or gastrointestinal cancer needed to meet in this study.

*Inclusion criteria:*
a. Over 18 years of age.
b. The patients were diagnosed with esophageal or gastrointestinal cancer by a general surgeon after pathological examination.
c. Have a certain ability to read words, be able to communicate orally and in writing.
d. Voluntary participation in the experiment.
e. No major events such as surgery during the experiment.
*Exclusion criteria:*
a. The communication is difficult, and the consciousness is not clear.
b. Suffer from other serious physical diseases.
c. Have a history of mental illness; take antipsychotic drugs or have recently stopped taking drugs for less than three months.
d. Other relevant mental and cognitive interventions are in progress.

### Measurement

The study conducted measurements of participants including preference, psychological, and physiological aspects to indicate the level of psychophysiological restoration. Psychological measurements included the Self-Rating Depression Scale (SDS), Perceived Restorativeness Scale (PRS), and the Positive and Negative Affect Scale (PANAS), while physiological measurements included HR, blood pressure (BP), electroencephalogram (EEG), and neutrophil-to-lymphocyte ratio (NLR). Many of these indicators were selected with the guidance of tumor-related attending doctors and the head nurses.

### Preference

Participants’ perceived preferences for landscape type were obtained by scoring on a seven-point Likert scale when viewing each panoramic photo by VR glasses. It is indicated that higher scores given by the participants signify a greater preference for the respective photo. The participants were encouraged to write down the reasons for the scoring, which was of great importance to further understand their landscape preferences.

### Psychological measurement

The level of self-report pressure of the participants was measured by the SDS (Shen et al., 2012), which had been widely used in the self-assessment of clinical depression (Liu et al., 2019). The scale consists of 10 positive items and 10 negative items, and it requires respondents to give scores according to the four occurrence frequencies, ranging from 1 to 4. The total score of the scale can be expressed by the sum of 20 items multiplied by 1.25. The threshold of the scale indicating depression is 53. The higher the scores were, the higher the degree of depression was.

The perceived restoration of respondents was measured by PRS (Hartig et al., 1997). It contains 16 items according to the attention restoration theory (ART): being away, fascination, coherence, and compatibility. The Likert-7 scale was used with a higher score representing a higher level of restoration potential.

The mood changes of respondents were determined by the PANAS (Watson et al., 1988). The scale consists of 20 items. Ten items indicated negative emotions (PANAS NEG), describing emotions such as nervous, scared, etc. The others indicated positive emotions (PANAS POS), describing emotions such as interested, excited, attentive, etc. The item scores consist of five degrees, from none to extremely. After the stimulation using VR panoramic photos, respondents were asked to fill out this scale to indicate mood changes.

The total Cronbach’s α coefficient of the scales is 0.824 for SDS, 0.819 for PRS, and 0.807 for PANAS, respectively (all >0.7), which means that the evaluation projects have high correlations and internal reliabilities are quite reliable.

### Physiological measurement and blood sampling

HR and BP were common physiological measurements indicating patients’ physical states in environmental psychological studies for their readily available data. HR could be regarded as one of the physiological responses to physiological stimulation and psychological stress (Reeves et al., 2019). BP is divided into diastolic blood pressure (DBP) and systolic blood pressure (SBP), which could be used to indicate the degree of relaxation of the body (Kulkarni et al., 1998).

Electroencephalogram (EEG) had been widely used in the interference study of external stimuli to respondents due to its non-intrusive and rapid collection of brain wave data (Chiang et al., 2017). It can fundamentally measure different aspects of the electrical activity of the brain to some extent. And the reliability of using EEG to measure users’ physiological responses in VR interventions has been proved in previous studies (Tarrant et al., 2018; Murphy and Higgins, 2019). The Alpha wave of the EEG is closely related to positive emotion and stress relief. Increased Alpha wave usually indicates relaxation (Klimesch, 1999; Fachner et al., 2013). Considering the previous studies showed that the increase of EEG alpha waves’ value can reflect the physiological relaxations experienced when one is exposed to natural features (Gao et al., 2019b), portable brain wave devices (NeuroSky, with the NeuroSky TGAM brain wave chip inside) were used to measure the changes of Alpha waves among participants to indicate their degree of restoration (Cacioppo et al., 2000). Through wireless devices and electrodes connected to the forehead, the brain wave data were transmitted to the computer in real time and displayed in numerical form.

The examinations of the blood samples were conducted to assess the hematological characteristics of the respondents. Neutrophil-to-lymphocyte ratio (NLR), which could reflect systemic inflammatory response and immune status, was selected as one of the physiological indicators (Jiang et al., 2016). Considering that inflammation plays a key role in the occurrence and development of cancer, it is speculated that it can be associated with the development of prognosis in a variety of tumors, including esophageal and gastrointestinal cancers (Murakami et al., 2019). Studies showed that NLR usually increased gradually as pathological stages progress, i.e., the lower its value, the better the survival rate and condition (Shibutani et al., 2013).

### Experimental design

The experiments were conducted in the laboratory after communication with the head nurse to ensure no external intervention occurred and consistent physical conditions were maintained throughout experiments. This experiment was conducted over the course of 1 week. After discussing the study and communicating with respondents, those who agreed to participate in the experiment received 100 RMB (approximately equal to $14.25) of rewards; participants would then conduct 3–5 interventions in a week depending on their physical and mental state in order to compare the number of interventions needed to achieve maximum restorative experience. The two groups had no significant difference in the number of interventions conducted (Table 2).

**Table 2:** Distributions of the participants among the different times of interventions in the intervention group and control group.

Group	Times of intervention (No. of the participants)		
	Three-time interventions	Four-time interventions	Five-time interventions
Intervention group	11	10	11
Control group	10	15	6
Significance of difference (Sig.)	95% C.I. (−12.17, 12.84), *T* = 0.11, *p* = 0.919		

Blood samples were collected before the first and after the last interventions. Considering the requests of patients and their families, no photos were taken during the experiment due to privacy.

Prior to the formal experiment, the purpose and process were introduced to the participants to decrease their nervousness and ensure their understanding of the experimental procedures. Participants were not allowed to communicate with each other until the experiment was complete. The pre-test stage included a questionnaire, physiological measurements, and an EEG baseline measurement. The questionnaire included basic information, SDS, and PANAS. Among them, the basic information section records the gender, age, educational degree, income, and living environment of the respondents. During the physiological measurements, the respondents sat in the laboratory for 3 min before the first measurement was taken; BP and HR were then measured twice using an electronic sphygmomanometer, and there was a 1-min interval between the two measurements. After that, the portable EEG electrode was placed onto participants’ foreheads for baseline measuring. Participants were asked to sit facing a white wall to temporarily exclude external visual stimuli. They were then asked to open their eyes and look at the wall for 1 min, and alternately close their eyes for another minute to determine their baseline brainwaves in order to identify the baseline of psychological stress before the experiment.

For visual stimulation, panoramic photos were displayed using the VR glasses. Each participant in the intervention group was asked to randomly view one selected panoramic photo for each category (BS, OGS, SOS, and CGS), for a total of four images each time. The participants in the control group were asked to view the photos of GrS only. Each photo lasted 1 min 20s and the total length of broadcast time was 5 min 20s. This time had been adjusted through preliminary experiments to ensure that the respondents could be fully immersed in each type of landscape without undue burden caused by prolonged exposure. The changes in brain waves would be recorded in real time when respondents in both groups viewed the panoramic photos.

**Figure 2:**
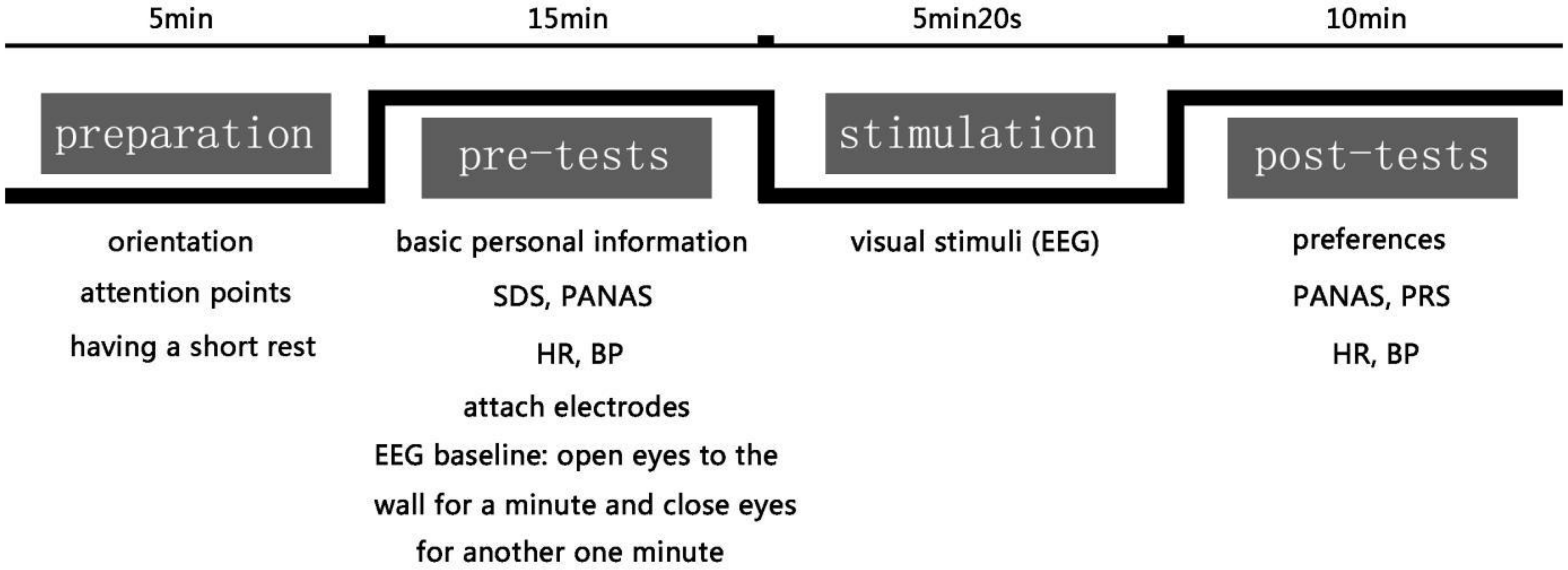
The study procedure within one experimental intervention and the corresponding time used within each stage.

During the post-tests, the respondents would give preference and PRS scores for each type of environment and fill out the PANAS once again. During this time, the BP and HR were measured twice again using an electronic sphygmomanometer with a 1-min interval between the two measurements (Figure 2). It is worth noting that the experimental procedure of the last experiment was the same as that of the first, while the other interventions only recorded the changes of brain waves during the viewing of the photos.

### Statistical analysis

The study used SPSS 17.0 (Statistical Package for the Social Sciences version 17.0) software to conduct the statistical analysis. First, the independent sample *T*-test was conducted on the demographic information of participants in the intervention group and the control group to examine whether a significant difference existed between the two groups, so as to ensure that the demographic information did not interfere with differences in experimental data between the two groups. Similarly, the initial levels of depression (SDS) in both groups were also tested to examine the differences between groups.

To understand the difference in preference of participants for the five landscape types, the study firstly used the independent samples *T*-test to analyze the difference of participants’ preferences between the intervention group (viewed different types of blue and green spaces) and control group (viewed gray space). And then, arithmetic means and ANOVA with *post hoc* tests were used to analyze the difference in preferences among the different types of blue and green spaces. The psychophysiological restoration potentials of the environmental spaces between the two groups during the pre-tests and post-tests were mainly examined by the paired *T*-test, using data collected from the PANAS, HR, and BP. At the same time, in order to test the differences in restoration levels between the two groups, an independent sample *T*-test was conducted to show the degree of the restoration of PANAS, HR, BP, and PRS. Groups were regarded as grouping variables, while the restoration differences before and after the experiment were regarded as the test variables. The arithmetic means and ANOVA with *post hoc* tests were conducted to show the restoration differences of PRS and EEG among different landscape types due to the expression of restoration ability and its significance.

The paired *T*-test was used to examine the differences in landscape preferences and psychophysiological restoration of patients in both groups under different intervention periods (times). Taking into account the external interference during the week-long experimental procedures, the study compared significant differences in the variation of psychophysiological indicators between the first and last experiments instead of the post-tests results only. In order to further explore the best intervention effect in a week and regarding the number of interventions as the classification criteria, the relaxation levels in brain waves within each intervention in the intervention group and the control group were expressed by calculating the mean values, respectively.

Goodness-of-fit of the models was assessed by Pearson’s Chi-square and deviance tests to ensure the models fit the data adequately.

## Results

The results showed that there were no significant differences in demographic information between the respondents in two groups, including gender (*p* = 0.54), age (*p* = 0.60), education degree (*p* = 0.57), income (*p* = 0.34), and living environment (*p* = 0.14). In addition, there was no significant difference between the two groups in the Self-Rating Depression Scale (SDS) (*p* = 0.92, mean value of the intervention group = 50.23, mean value of the control group = 50.52).

### The most preferred type of environment within cancer patients based on comparative analysis of intervention group and control group

**Figure 3:**
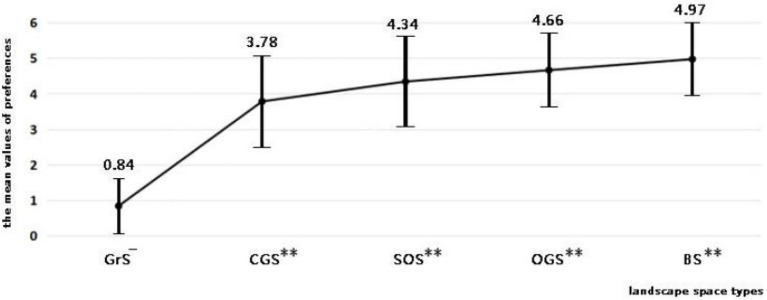
Mean values of participants’ preferences for different landscape space types. I. GrS refers to the gray space. CGS refers to the closed green space landscape. SOS refers to the semi-open green space landscape. OGS refers to the open green space landscape. BS refers to the blue space landscape. II. — refers to the reference landscape. ^**^ refers to the significant difference between the landscape and the reference landscape (*p* < 0.01).

The results of the independent sample *T*-test showed that there was a significant difference in preferences between the intervention group and control group (*p* < 0.01). The arithmetic mean of the preference level of patients for the BS and GS in the intervention group was 4.44, while that for GrS in the control group was 0.84. In addition, there were also significant differences in the preferences of participants in the intervention group for BS and GS (*p* < 0.01). Results showed that BS was the most significantly preferred landscape type (mean values = 4.97 ± 1.00), followed by OGS (mean values = 4.66 ± 1.00), SOS (mean values = 4.34 ± 1.23), and CGS (mean values = 3.78 ± 1.26; Figure 3).

### The differences in psychological and physiological restoration of cancer patients in two groups among different types of environment

It was found that for both the intervention group and the control group, the positive emotions (PANAS POS) of the participants increased significantly after the visual stimuli, and the negative emotions (PANAS NEG) decreased significantly. Through the comparison between the two groups, their changes in positive emotions were significantly different, and the restoration level of the intervention group was significantly higher than that of the control group. Although there was no significant difference in negative emotions, the landscapes in the intervention group greater reduced negative emotions than the gray landscape in the control group (Table 3). For PRS, the results of the arithmetic means and ANOVA with *post hoc* tests showed that the BS had the significantly strongest potential to increase perceived restorativeness of the respondents, followed by OGS, SOS, and CGS, with the GrS having the least potential.

**Table 3:** The results of the *T*-test and ANOVA (mean values ± standard deviation) concerning the effects of one visual stimulation on psychological and physiological restorative indicators of patients and the differences between the two groups.

	The intervention group			The control group		
	Pre-tests	Post-tests	MD	Pre-tests	Post-tests	MD
PANAS POS^a^	25.06 ± 6.84	30.88 ± 6.13	−5.82 ± 5.12^b^	19.71 ± 4.01	22.55 ± 5.19	−2.84 ± 4.82^b^
PANAS NEG	15.53 ± 5.30	11.28 ± 2.62	4.25 ± 3.97^b^	16.94 ± 5.27	12.97 ± 2.20	3.97 ± 4.15^b^
PRS	68.16 ± 10.50 (OGS)			12.39 ± 6.46 (GrS)		
	66.47 ± 15.40 (SOS)					
	56.78 ± 17.08 (CGS)					
	78.22 ± 14.41 (BS)					
	*Post hoc*: BS^b^ > OGS^b^ > SOS^b^ > CGS^b^ > GrS^—^					
Heart rate (HR)	75.08 ± 12.81	72.39 ± 13.54	2.69 ± 4.41^b^	75.89 ± 13.47	74.98 ± 11.90	0.91 ± 4.78
Diastolic blood pressure (DBP)	70.86 ± 10.80	71.31 ± 9.89	−0.45 ± 4.99	75.32 ± 10.89	74.10 ± 9.70	1.22 ± 3.62
Systolic blood pressure (SBP)	113.63 ± 16.26	114.58 ± 14.23	−0.95 ± 8.15	121.71 ± 19.06	119.18 ± 16.94	2.53 ± 7.49
EEG (*10^−2^)	6.61 ± 1.33 (open eyes)			8.82 ± 4.88 (open eyes)		
	12.39 ± 7.02 (closed eyes)			9.40 ± 5.33 (closed eyes)		
		7.36 ± 1.90 (OGS)			8.17 ± 4.16 (GrS)	
		6.83 ± 1.36 (SOS)				
		6.94 ± 1.37 (CGS)				
		6.86 ± 1.47 (BS)				
	*Post hoc*: closed eyes^—^ > OGS^b^ > CGS^b^ > BS^b^ > SOS^b^ > open eyes^b^			*Post hoc*: closed eyes > open eyes > GrS		

I. MD refers to the mean difference between the pre-tests and the post-tests. II. GrS refers to the gray space. CGS refers to the closed green space landscape. SOS refers to the semi-open green space landscape. OGS refers to the open green space landscape. BS refers to the blue space landscape. III. — refers to the reference landscape.

a Refers to the significant difference of MD between the intervention group and the control group.

b Refers to the significant difference between the landscape and the reference landscape (*p* < 0.01) or the significant difference between the pre-tests and post-tests. PANAS POS.

For the HR and BP indicators, except for the significant reduction of the HR in the intervention group during the post-tests (*p* < 0.01), there were no other significant differences between the two groups. For EEG, based on the arithmetic means and ANOVA with *post hoc* tests, the participants in the intervention group presented a greater trend of relaxation after the visual landscape stimuli than the open eye stage during the baseline measurement. The EEG alpha waves were the highest when the respondents’ eyes were closed in the intervention group, and mean values of relaxation in the OGS, CGS, BS, and SOS were higher than that in the open eye stage. These differences were significant. For the control group, the EEG alpha wave value was the highest in the closed eye stage, followed by the open eye stage, and finally the visual stimuli stage (the gray space, GrS; Table 3).

### The timeliness of psychophysiological restoration within cancer patients under multiple-time intervention in a week

Considering the large variation in the intervals of blood collection, the experiment did not conduct further analyses of the NLR level of participants in order to avoid result bias. While the results showed that the self-reported depression level of both groups decreased significantly after 1 week of visual stimulation intervention (*p* < 0.01), the level of perceived restoration (PRS) and landscape preference for the gray space was significantly decreased. Although the levels of the perceived restoration (PRS) and landscape preference also decreased after viewing the green and blue spaces, the changes were not significant. Moreover, the preference and the perceived restoration of respondents for the blue scape were slightly increased, and slightly reduced in the green space landscape. The level of negative emotion reduction by landscape stimulation was significantly different after 1 week of intervention (*p* < 0.01), and the ability of landscape to reduce negative emotions decreased after 1 week of intervention. In addition, compared with the first experiment, there were no significant differences in the restoration of physiological indicators in both groups after several interventions of landscape stimuli during a week. After 1 week of intervention, the effects of blood pressure reduction and positive emotion promotion of the intervention group were slightly improved, and the ability to decrease the HR was slightly reduced, while the opposite was true for the control group. With the increase in intervention times, the ability of different landscapes to sooth mental stress (EEG) was weakened (Table 4).

**Table 4:** The results of the paired *T*-test examining the various psychophysiological indicators between the first and the last experiments (one-week period).

	SDS		PRS		PANAS POS^a^		PANAS NEG^a^	
	Intervention	Control	Intervention	Control	Intervention	Control	Intervention	Control
First experiment	50.23	50.52	67.41	12.39	5.81	2.84	−4.25	−3.97
Last experiment	41.87	44.07	67.08	9.39	6.66	1.61	−2.19	−1.45
Significance of difference (Sig.)	<0.01	<0.01	n.s.	0.04	n.s.	n.s.	<0.01	<0.01
	HR^a^		Diastolic blood pressure (DBP)^a^		SBP^a^		EEG (*10^−2^)	
	Intervention	Control	Intervention	Control	Intervention	Control	Intervention	Control
First experiment	−2.69	−0.90	0.45	−1.23	0.95	−2.53	7.00	8.17
Last experiment	−1.67	−1.92	−0.17	0.13	−1.47	−2.00	7.63	7.48
Significance of difference (Sig.)	n.s.	n.s.	n.s.	n.s.	n.s.	n.s.	n.s.	n.s.
		Preferences						
		Intervention				Control		
First experiment		4.44				0.84		
Last experiment		4.30				0.42		
Significance of difference (Sig.)		n.s.				<0.01		

a I. means that the study tested the significant differences of the variations in the first and the last experiments instead of the differences of the psychophysiological result only. II. n.s. means that there is no significant difference.

**Figure 4:**
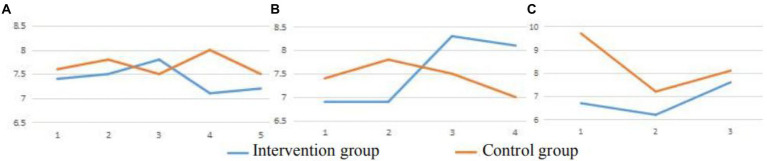
Mean values of Alpha brain waves. **(A)** The mean values of Alpha brain waves across five interventions. **(B)** The mean values of Alpha brain waves across four interventions. **(C)** The mean values of Alpha brain waves across three interventions.

The mean values of alpha brain waves showed that through repetitive visual interventions, the relaxed state of participants in the intervention group inclined to the highest level until the third intervention in a week. This indicates that three times a week may be the most suitable frequency to result in the highest level of relaxation. It may be better for cancer patients to be in contact with the natural environment three times a week rather than multiple times (Figure 4).

## Discussion

In order to account for the fact that cancer patients were rarely studied in previous research, and for a lack of comparison of preferences and recovery effects between long-term and short-term experiments, the current study mainly examined the preferences of patients with esophageal and/or gastrointestinal cancer for five types of environment and the differences in their recovery in the short term and long term.

### The preferences of different types of landscape

According to the arithmetic means and ANOVA with *post hoc* tests, the participants had the highest preference for BS, followed by GS, while GrS received the lowest preference. This suggests that compared with the gray space common in urban settings, natural environments of blue and green spaces are generally welcomed and appreciated by cancer patients, which is in line with previous conclusions (Jorgensen and Anthopoulou, 2007; Ibarra et al., 2017). Humans evolved in nature, and they prefer nature no matter the age and culture (Kaplan and Yang, 1990; Meidenbauer et al., 2019). This preference also applies to cancer patients, and has also been found to improve their cognitive performance (Ulrich et al., 1991). Blue space can give cancer patients a sense of being far from urban life by providing a serene environment and creating opportunities to meet their hydrophilic nature.

For the green space, it was found that the open green space was the most preferred, followed by the semi-open green space and the closed green space. This is in line with previous studies as well (Giergiczny et al., 2015; Ebenberger and Arnberger, 2019). However, varying opinions do exist. Wang et al. (2019) found a positive correlation between plant number and preference, while Gao et al. (2019b) found that semi-structural green space was the most popular due to the tendency of untidy feelings that complex vegetation structure provides and the lack of security in open space. Some reasons for the differences in findings between this study and previous studies follow. On the one hand, this study used VR with panoramic 3D photo intervention, which is different from the traditional field survey and photo elicitation experiments. The immersion of VR can not only reflect the real situation on demand indoors, but also possibly shields the interference of other factors, which could be of positive significance to the results to reflect the true preference of the participants. On the other hand, preference is not only related to the features of landscape, but also to human attributes (Maulan and Miller, 2006). Cancer patients with activity inconvenience needed a greater sense of security and social care, as well as opportunities for appropriate activities. In this study, the open green spaces provided cancer patients with visual accessibility and therefore brought a sense of security, just as one participant mentioned “I can see everything with a wide view here.” Compared with the complex structure of other types of green spaces, open green space does not require much attentional or cognitive effort because ground vegetation is one of the most important elements affecting landscape preferences (Nielsen et al., 2012). The tidy short-cut lawn may provide the patients with a visible sense of management and social care, and was described as “the lawn is trimmed and tidy” and “the lawn seems comfortable.” Furthermore, research showed that open green spaces are more conducive to active recreational activities including physical exercise (Boxall and Macnab, 2000; Eriksson et al., 2012). Therefore, the open green space in this study might meet the needs of cancer patients and thus was generally welcomed.

### The differences in psychophysiological restoration of cancer patients among different types of environment

The study found that the positive effect of nature on psychological health was significantly higher than that of gray space. This may be due to the fact that natural space gives cancer patients a sense of consistency, e.g., harmony and relatedness to themselves (Sevenant and Antrop, 2009; Peschardt and Stigsdotter, 2013). Most participants in this study lived in rural areas. They mentioned that the natural landscape was similar to the environment in which they lived, and it invoked a pleasant feeling. It is in line with their instinctive judgment that natural space is suitable for survival and prosperity, thus achieving psychological well-being (Ulrich, 1977; Kaplan et al., 1998). It is worth noting that the psychological indicators of the patients in the gray space, such as emotion and perceived restoration, also showed a certain improvement. This might indicate that cancer patients can gradually calm themselves during daily life without any intervention, but the natural environment can increase the ability of psychological restoration and emotional recovery. In addition, the results of this study showed that the participants had the highest level of perceived restoration in the blue space, followed by green space, and finally in the gray space, which is exactly the same as the preference trend. The blue space is not only the most popular environment, but also the most beneficial to the improvement of the patients’ perceived restoration, followed by the open green space (Appleton, 1975). These spaces seem to be quite different from the common landscapes of daily urban space and can give cancer patients the feelings of being far away from the city and medical treatment. The beautiful bodies of water and the manicured large lawn are examples of the prospect-refuge theory, which describes a landscape that is open and without place for predators to hide (Appleton, 1975). These environments are popular and have restorative functions. Moreover, in line with the attention restoration theory (ART), the low complexity of blue space and open green space can provide attractive scenery while reducing energy consumption to alleviate mental fatigue (Joye and van den Berg, 2011), helping the cancer patients to deal with natural characteristics more easily (Joye et al., 2016), and therefore reducing the consumption of psychophysiological energy.

Interestingly, this study found that despite the preferences and benefits of psychology, the recovery of physiological indicators was not as significant. This is probably due to the fact that the change in physiological indicators was not as significant as psychological indicators, so that 5 min and 20s of landscape stimulation could not be adequate enough to produce a significant physiological response. Chang et al. (2008) found that the EEG alpha waves of a simulated landscape with a restorative function were increased compared to other simulated landscapes. As an indicator of relaxation, alpha brain waves showed that patients’ relaxation was the highest when they closed their eyes, even higher than in the landscape stimulation stage. This is somewhat inconsistent with previous studies (Tyrväinen et al., 2014), although closed eyes can improve the degree of relaxation of the body and mind. Considering no work was assigned to the cancer patients in the eyes closed stage, the visual stimuli and the viewing of the white wall might have given the participants a sense of burden and thus was not conducive to their relaxation. This might be attributed to the difference between cognitive load and no cognitive load, as it is only when the eyes are open that people can infer a degree of cognitive workload (Onishi and Hagawara, 2017). Compared with the eyes-closed conditions, other brainwaves could be activated separately during the eyes-open conditions (Barry et al., 2007). Therefore, further examination and consideration is required in choosing eyes-closed conditions as baseline conditions and physiological indicators in future studies. However, the relaxation level in the landscape stimuli stage was higher than that of patients in the eyes open stage, which suggested that natural landscape can bring certain physiological and psychological benefits compared with the general state. After all, since a variety of visual stimuli filled the patients’ time when their eyes were open during the average day (Onishi and Hagawara, 2017), they can receive mental restoration through nature interventions, which indicated that VR can be applied as a useful and feasible tool for restorative experience with cancer patients.

### The timeliness of psychophysiological restoration

This study proved that repetitive natural landscape interventions within a week could enhance the physical and psychological restoration potential of patients. Previous studies have also examined the intervention terms in different landscapes, but instead focused only on the length change of one experiment. For example, Ulrich et al. (1991) demonstrated the positive effect of nature on physiological indicators through a 10-min nature intervention. Furthermore, the participants did not focus on or include cancer patients, or include varying periods of interventions. Shanahan et al. (2016) proposed the benefits of a 30-min natural environment for hypertensive patients, but the impact of time spent in nature on mental health was relatively unexplored for cancer patients (Trostrup et al., 2019). It was demonstrated that long-term exposure to natural space had more advantages than short-term exposure, which might be due to the accumulation of natural rehabilitation benefits. Three times a week proved to be the best intervention frequency for cancer patients to relax, indicating that this frequency of intervention met their relaxation requirements without producing a negative burden and emotion over time, such as boredom. Based on these important findings and considering that the cooperation of cancer patients (physical condition, objective influence, etc.) is also necessary for a long-term study, exploration is still required to determine how long this effective rehabilitation can last in the future.

### Limitations and implications for further research and landscape plans

To a certain extent, although this study filled a gap existing in previous studies of landscape preference on the psychophysiological restoration of cancer patients within long-term interventions, it also has some inadequacies. First, although considering the high incidence and mortality of esophageal and gastrointestinal cancer diseases (Anderson et al., 2019; Zhao and Lim, 2020), other kinds of cancer patients were not included. Demographic characteristics could also affect the experimental results of cancer patients (Holm et al., 2012), so subsequent studies could focus on cancer patients with specific demographic characteristics. Second, more quantitative measures of immune indicators are needed. One of the important positive links between nature and health is the increase in immune ability (Kuo, 2015). However, there has not been a study conducted to explicitly link green and blue spaces in cities to improvements of the human immune system. Third, perception and preference are the result of multiple sensory combinations (Subiza-Pérez et al., 2019). Future experiments can incorporate other senses, such as audio, tactus, and olfaction, which might change the results of the study. Fourth, as for the experimental design, HR were only measured in pre-and post-test stages in our study. It is better and more accurate to conduct a real-time monitoring during the whole experiment in further research. Moreover, participants’ prior experience of using VR equipment should be asked in further study, since it may relate to their adaptation to VR and affect the results.

## Conclusion

This paper explored the esophageal and gastrointestinal cancer patients’ preference and restoration potential in various landscapes under different intervention times by VR. Some main conclusions were obtained. First, the blue and green spaces were more popular among cancer patients than the gray space (hospital environment). They were also beneficial to the psychological health (e.g., emotion and perceived restoration) and recovery of some physiological indicators (e.g., HR). Second, the cancer patients showed a high degree of relaxation when they had nature interventions in spite of the peak level of relaxation during the eyes-closed period in the EEG baseline measuring stage. Finally, three times a week seems to be the most suitable frequency for cancer patients to be exposed to nature for psychological health. These conclusions provide a theoretical basis and interdisciplinary guidance for the construction of cancer-patient-oriented environments. For example, blue space and open green space could not only satisfy the preference of patients, but also be conducive to their psychophysiological recovery. More attention should be thus paid to the appropriate increase of blue space and the use of open green space in the improvement of hospital and rehabilitation community landscapes. In addition, for patients whose movements are restricted, they can receive the benefits of nature through viewing natural environments through their hospital windows, while for those who are completely incapacitated, VR may be utilized to conduct natural interactions three times a week to improve physical and mental restoration and reduce the stress of medical treatments such as chemotherapy.

## Data availability statement

The original contributions presented in the study are included in the article/supplementary material, further inquiries can be directed to the corresponding authors.

## Ethics statement

The study was conducted in accordance with the Declaration of Helsinki, and the protocol was approved by the Ethics Committee of the College of Landscape Architecture and Arts, Northwest A&F University. Written informed consent to participate in this study was provided by the patient/participants.

## Author contributions

All authors listed have made a substantial, direct, and intellectual contribution to the work, and approved it for publication. All authors contributed to the article and approved the submitted version.

## Funding

This research was funded by the National Natural Science Foundation of China [grant no. 31971720], the Fundamental Research Funds for the Central Universities [grant no. 2452019175], the Science and Technology Innovation Program of Shaanxi Academy of Forestry [grant no. SXLK2021-0216], the Key Research and Development Program of Xianyang [grant no. 2021ZDYF-SF-0022], and the Scientific Research Cooperation Agreement Project of the Xianyang Forestry Bureau [grant no. 20211221000007].

## Conflict of interest

The authors declare that the research was conducted in the absence of any commercial or financial relationships that could be construed as a potential conflict of interest.

## Publisher’s note

All claims expressed in this article are solely those of the authors and do not necessarily represent those of their affiliated organizations, or those of the publisher, the editors and the reviewers. Any product that may be evaluated in this article, or claim that may be made by its manufacturer, is not guaranteed or endorsed by the publisher.
